# *Chelidonium majus* L.-containing gel may improve diabetic wound healing by modulating MMP-2, MMP-9, and collagen levels

**DOI:** 10.55730/1300-0152.2757

**Published:** 2025-04-21

**Authors:** Kaan KALTALIOĞLU, Elif Naz GÜRSOY, Kanuni Barbaros BALABANLI, Zeki AYTAÇ, Şule COŞKUN CEVHER

**Affiliations:** 1 Programme of Occupational Health and Safety, Vocational School of Espiye, Giresun University, Giresun, Turkiye; 2 Department of Biology, Faculty of Science, Gazi University, Ankara, Turkiye

**Keywords:** *Chelidonium majus*, diabetic wound healing, MMPs, phytotherapy, oxidative events

## Abstract

**Background/aim:**

Delayed wound healing in diabetic patients is a significant complication that reduces quality of life, prompting the continuous investigation of new therapeutic agents. This study designed to explore the dose-dependent effects of different parts of *Chelidonium majus* L. (CM), a medicinal plant traditionally used for skin disorders, on diabetic skin wounds.

**Materials and methods:**

In diabetic rats, full-thickness excisional wounds were formed. CM-containing gels (aerial parts at 3%, 6%, 9% and root at 0.5%, 1%, 1.5%) were developed and applied to the wounds. After the treatment period, the rats were sacrificed, and wound healing activity was assessed macroscopically, histopathologically, and biochemically.

**Results:**

The CM-containing gels (aerial parts or root) accelerated wound closure and increased collagen, glutathione (GSH), and ascorbic acid (AA) content. Additionally, these gels reduced oxidative stress markers, and interleukin-1β (IL-1β) and IL-13 levels, while modulating the activities of matrix metalloproteinase-2 (MMP-2) and MMP-9.

**Conclusion:**

CM accelerates the healing process by increasing antioxidant capacity and modulating MMP activity, and it may have dose-dependent effectiveness in diabetic wound management.

## 1. Introduction

Diabetes mellitus (DM) is a metabolic disease generally characterized by excessive blood glucose levels and affects millions of individuals worldwide (Chen et al., 2023). One of the important complications of DM is that it causes various vascular disorders leading to impaired healing of wounds and ulcers (Agyare et al., 2016). The delayed healing of diabetic wounds is a complex phenomenon, influenced by various factors including hyperglycemia, neurological and vascular complications, decreased production of growth factors, impaired macrophage function, reduced angiogenesis, and collagen synthesis (Shenoy et al., 2016; McLaughlin et al., 2021; Wu et al., 2022). In addition, wounds in diabetic patients are characterized by excessive inflammation, wound dehiscence, an increased risk of infection, decreased angiogenesis, imbalances in proinflammatory and antiinflammatory agents, and oxidative stress (Thandavarayan et al., 2015; Patel et al., 2022). Developing effective treatment strategies to reverse these abnormal activities is extremely important for diabetic individuals. The use of various substances for topical application is being investigated as a therapeutic approach for diabetic wounds.

Medicinal plants are agents that have been used since ancient times to treat many diseases and disorders in medical care. They are preferred over synthetic agents, partly because they are cheaper, more readily available and believed to have fewer side effects. *Chelidonium majus* L. (CM, also called greater celandine), one of the most significant medicinal plants in the family Papaveraceae, is a perennial herbaceous plant that can be found in the majority of Europe, North Africa and Western Asia such as Italy, France, Morocco, Armenia, Türkiye and Azerbaijan (Staniak et al., 2019; Zare et al., 2021). CM contains a complex mixture of alkaloids (e.g. berberine, chelidonine, sanguinarine), flavonoids (e.g. rutin, quercetin) and phenolic acids (e.g. chlorogenic and gallic acid) (Nawrot et al., 2016; 2021). It has antiviral, antitumor, antimicrobial, anti-inflammatory, and hepatoprotective effects (Och et al., 2019; Zare et al., 2021), and is used folklorically in various countries for the relief or alleviation of skin disorders, bronchitis, jaundice, and diseases or disorders of the digestive system, eye, and liver (Staniak et al., 2019; Zare et al., 2021).

Impaired wounds have a negative influence on the psychological and social life of patients, in addition to health concerns. Diabetic wound healing often relies on synthetic drugs and advanced techniques such as bioactive dressings and negative pressure wound therapy, which aid infection control and tissue repair, but face challenges such as high cost and side effects. Medicinal plants such as CM offer a natural alternative due to their antioxidant, antiinflammatory and antimicrobial properties. Rich in alkaloids and flavonoids, CM promotes tissue regeneration and modulates inflammation (Borges et al., 2024; Gürsoy et al., 2024). Formulation of CM as a gel ensures localized delivery and enhances its therapeutic efficacy, making it a promising candidate for diabetic wound care. The objective of this work is to scientifically explore the dose-dependent effects of the aerial and root parts of CM, which have been reported in ethnobotanical studies to be useful in wound treatment, on the diabetic healing process, and to determine the effects on oxidative events during wound tissue repair.

## 2. Materials and methods

### 2.1. Plant material and extraction

CM was collected from rocky areas (1200 m a.s.l.) between Kızılcahamam and Çamlıdere in Ankara province (Türkiye). The collected plant samples were identified by Prof. Dr. Zeki AYTAÇ (Gazi University Herbarium, GAZI 29835). The aerial parts and roots of the plants were separated, dried in the shade, and ground in a commercial Waring blender (Waring Commercial, Torrington, CT, USA). The aerial parts (10 g) or roots (10 g) were extracted with methanol in a Soxhlet device. The extracts were filtered using Whatman paper (Cytiva, Marlborough, MA, USA). A rotary evaporator was used to concentrate the methanolic extracts.

### 2.2. Topical gel formulation

First, carbopol (0.5 g) and glycerine (7 g) were mixed, followed by the gradual addition of distilled water (5 g). Separately, isopropyl alcohol (20 g) and carbopol (0.5 g) were mixed with distilled water (5 g), then combined with the first mixture. The solution was stirred at 25 °C until the carbopol dissolved. Next, 3 g of CM extract (aerial part or root) was mixed with distilled water (10 g) and added. Subsequently, triethanolamine (3.5 g) dissolved in water (5 g) was added. Distilled water was then added to bring the total volume to 100 g, and the mixture was stirred until gelation was complete and homogenized, forming a 3% extract gel (Ayla et al., 2019). Gels of other concentrations (aerial parts at 3%, 6%, 9% and root at 0.5%, 1%, 1.5%) were prepared similarly, adjusting for plant part and dose. Before application, animals were weighed, and gel dosages (e.g., 30 mg/kg/day) were administered accordingly.

To determine the appropriate doses for the topical application of the plant extract in animals, we considered our preliminary research, oral application studies (Lenfeld et al., 1981; Psotová et al., 2006), and the average use in humans (Benigni et al., 1962; Mazzanti et al., 2009), since there were no similar topical application studies involving the same plant in animals. In addition, we aimed to clarify this situation by using more than one dose in our study.

### 2.3. Experimental design, diabetes induction, and wound creation

The study was authorized by Local Animal Experiments Ethics Committee (Gazi University, G.U.ET-21.051). Table 1 shows the random assignment of 54 Wistar albino rats (male, 200–250 g) into nine groups. The animals were provided with rat chow and water, and housed individually with a natural daylight cycle. To induce diabetes, a single dose of streptozotocin (STZ) (55 mg/kg, i.p.) in a sodium citrate buffer (pH 4.5) was administered. After 3 days, rats with blood glucose levels of ≥ 250 mg/dL were sorted as diabetic (Kaltalioglu, 2023). Under anesthesia (xylazine and ketamine), the rats’ dorsal skin was shaved, and a 6 mm punch was used to create six full-thickness excisional wounds. The gels were administered topically once a day. In the positive control group, Madecassol (Bayer Türk, İstanbul, Türkiye) was used as the reference treatment (Ayla et al., 2019; Kaltalioglu, 2023), whereas the negative control group received no therapy. On day 7, the rats were anesthetized and sacrificed; skin samples were harvested and kept at −80 °C until the test.

### 2.4. Macroscopic and histopathological evaluation

**Figure 1:**
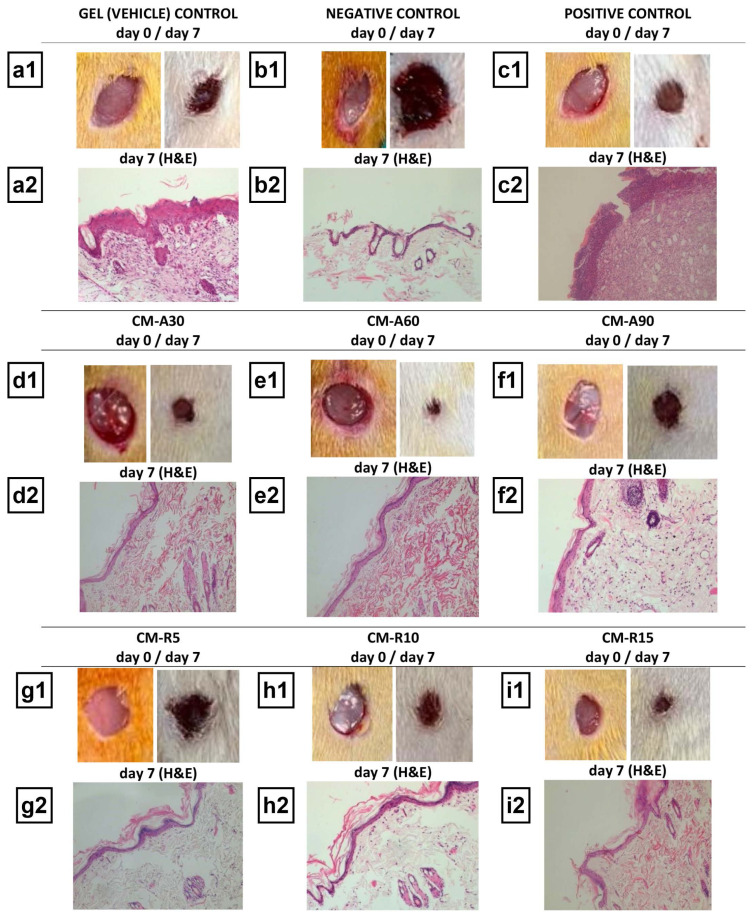
Macroscopic and histopathological evaluation of wound healing in the control and CM-containing gel-treated groups. a1–i1) Macroscopic views of the gel (vehicle) control, negative control, positive control, CM-A30, CM-A60, CM-A90, CM-R5, CM-R10, and CM-R15 groups, respectively. a2–i2) Corresponding histopathological examinations for the same groups.

The first and seventh days of the healing process were photographed with a digital camera (Canon Inc., Tokyo, Japan) (Figure 1), and the wound closure rates (WCRs) were then calculated using the previously described formula (Kaltalioglu, 2023), based on measurements made with the ImageJ program (NIH, Bethesda, USA). The tissue samples were embedded in paraffin and preserved in 10% formalin solution after surgery. After histological processing, 5 μm thick skin sections were stained with haematoxylin and eosin (H&E). Histopathological alterations in wound tissue sections were scored and computed according to the previously published method (Galeano et al., 2006).

### 2.5. Determination of MMP-2, MMP-9, AA, and collagen levels

The MMP-2 and MMP-9 levels in wound samples were measured using Rat MMP-2 and MMP-9 ELISA kits (SunRed Biotechnology Co. Ltd., Shanghai, China) following the manufacturer’s instructions. AA levels were determined spectrophotometrically at 515 nm (Berger et al., 1989). Wound samples were homogenized in perchloric acid (HClO_4_) with ethylenediaminetetraacetic acid (EDTA) on ice, centrifuged at 15,000 g, and the supernatants collected. The samples were mixed with a color reagent, incubated, and then H_2_SO_4_ was added.

### 2.6. Determination of TBARs, GSH, NOx, and PC levels

The thiobarbituric acid reactive substances (TBARs) concentration was measured spectrophotometrically at 535 nm (Casini et al., 1988), by homogenizing tissue samples in trichloroacetic acid, centrifuging them, and reacting the supernatant with thiobarbituric acid at 100 °C. GSH levels were determined by reacting homogenized samples with Na_2_HPO_4_ and 5,5-dithiobis-2-nitrobenzoic acid (DTNB), then measuring absorbance at 412 nm (Aykaç et al., 1985). Nitric oxide metabolite (NOx) levels were measured using the Griess reaction (Miranda et al., 2001), with homogenized tissues centrifuged and treated with sodium hydroxide (NaOH) and vanadium trichloride, using sodium nitrite as a standard. Protein carbonyl (PC) levels were assessed by reacting 2,4-dinitrophenylhydrazine (DNPH) with protein carbonyl groups, and measuring absorbance at 370 nm for PC and at 280 nm for total protein (Levine et al., 1990).

### 2.7. Determination of IL-1β, IL-13, and TNF-α levels

The levels of IL-1β, IL-13, and TNF-α in the collected serum samples were analyzed using ELISA kits (SunRed Biotechnology Co. Ltd., Shanghai, China) according to the manufacturer’s instructions.

### 2.8. Statistical analysis

Statistical significances across groups were analyzed using the analysis of variance (ANOVA) post hoc Tukey test (Prism 10 software; GraphPad Software LLC., San Diego, CA, USA). Differences were considered statistically significant when p values were less than 0.05. The data were provided as the mean ± SD.

## 3. Results

### 3.1. Macroscopic and histopathological evaluation

**Figure 2:**
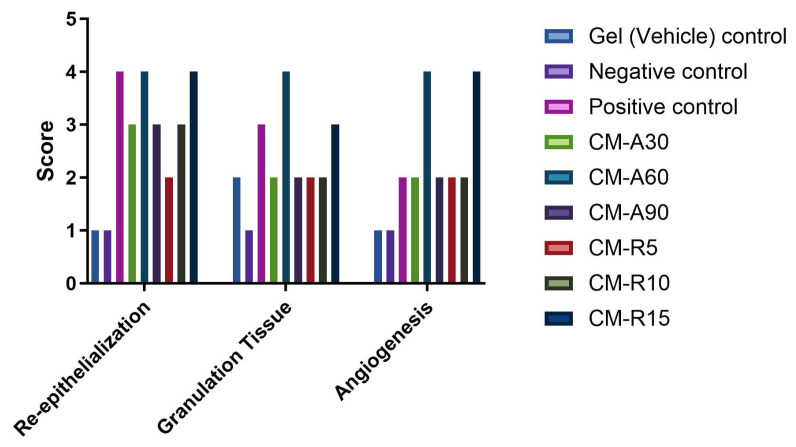
Histopathological scoring of wound tissue samples from all experimental groups.

The highest WCRs were observed in CM-A60 (79.31%) and CM-R15 (78.82%) groups, while the lowest WCRs were observed in the negative control (33.05%) and vehicle control groups (35.71%) (Table 2). Histopathological analysis of wound tissue sections (Figure 1) indicated that both the negative control (group II) and gel (vehicle) control (group I) groups failed to reestablish an intact epidermal barrier. These groups exhibited oedema, bleeding, reduced collagen content, accumulation of unhealthy granulation tissue, and presence of inflammatory cells. Conversely, the groups treated with CM-containing gels (groups IV–IX) and the positive gel (group III) demonstrated enhanced vascularization, increased presence of fibroblast cells and collagen fibrils, improved reepithelialization, and formation of skin appendages. Specifically, the CM-A90 group (group VI) showed reduced granulation and inflammation, while the CM-A60 group (group V) exhibited dense hair follicles, sebaceous glands, and collagen accumulation. Additionally, the groups treated with CM-containing gels (groups IV–IX) and the positive gel (group III) displayed superior histopathological scores compared to the negative control group, as illustrated in Figure 2.

### 3.2. MMP-2, MMP-9, AA, and collagen levels

**Figure 3:**
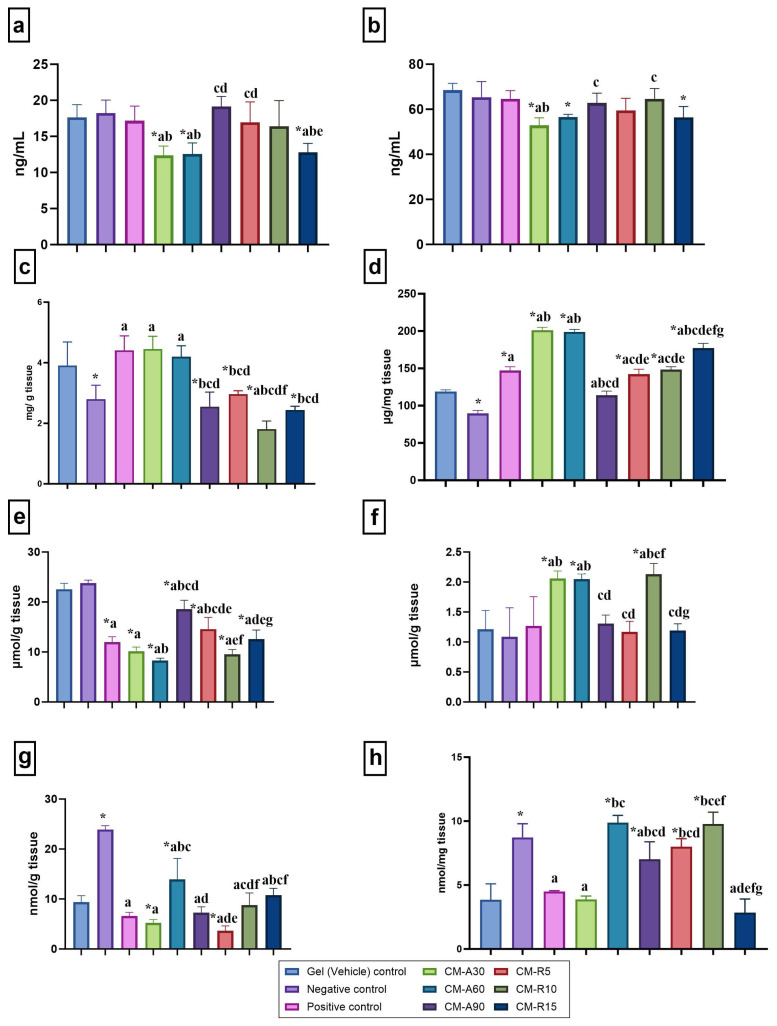
Oxidative stress and protein structure parameters in wound tissue samples. Statistical significance is indicated as follows: * compared to the gel (vehicle) control group (p < 0.05); ^a^ compared to the negative control group (p < 0.05); ^b^ compared to the positive control group (p < 0.05); ^c^ compared to the CM-A30 group (p < 0.05); ^d^ compared to the CM-A60 group (p < 0.05); ^e^ compared to the CM-A90 group (p < 0.05); ^f^ compared to the CM-R5 group (p < 0.05); and ^g^ compared to the CM-R10 group (p < 0.05). Measured parameters: a) MMP-2, b) MMP-9, c) ascorbic acid (AA), d) collagen, e) TBARs, f) GSH, g) NOx, and h) protein carbonyl (PC) levels.

According to Figure 3a and 3b, the negative control, the positive control, and the gel (vehicle) control groups had similar MMP-2 and MMP-9 concentrations. CM-A30, CM-A60, and CM-R15 gel applications significantly decreased MMP-2 levels compared to the negative and positive control groups (p < 0.05) (Figure 3a). Additionally, CM-A30 gel application significantly decreased MMP-9 levels compared to the negative and positive control groups (p < 0.05) (Figure 3b). Figure 3c shows that CM-A30 and CM-A60 gel applications increased the AA levels relative to the negative control (p < 0.05), while CM-R10 gel application decreased them. CM-containing gel applications (Group IV–IX) resulted in significantly higher collagen levels than the negative control group (p < 0.05) (Figure 3d).

### 3.3. TBARs, GSH, NOx, and PC levels

Following the application of CM-containing gels (Groups IV–IX), notable changes were observed in the measured antioxidant markers relative to the other groups. Specifically, TBARs levels were significantly lower in all CM-treated groups compared to both the negative control and the gel (vehicle) control group (p < 0.05), with CM-A60 exhibiting the most pronounced decrease (Figure 3e). In terms of antioxidant capacity, GSH levels were significantly elevated in the CM-A30, CM-A60, and CM-R10 groups compared to the negative control (p < 0.05) (Figure 3f). Furthermore, these groups also showed significant increases compared to the positive control group (p < 0.05). The CM-R10 group exhibited the highest GSH levels, which were significantly higher than those in CM-A90 and CM-R5 groups (p < 0.05) (Figure 3f). NOx levels were also significantly reduced in all CM-treated groups compared to the negative control (p < 0.05) (Figure 3g). Additionally, NOx levels in the CM-A60 group were higher than those in the other treatment groups, although still substantially lower than in the negative control group. The CM-A30, CM-A90, and CM-R15 groups had substantially lower PC levels than the negative control (p < 0.05) (Figure 3h). Compared to CM-A90, the CM-A60 group showed higher PC levels, whereas the CM-A30 group showed lower PC levels (p < 0.05). Furthermore, PC levels in the CM-R15 group were lower than in all other CM-treated groups except CM-A30 (p < 0.05).

### 3.4. IL-1β, IL-13, and TNF-α levels

**Figure 4:**
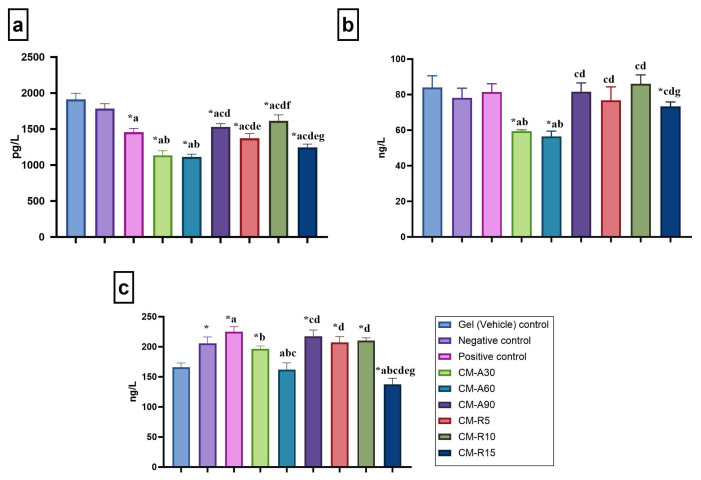
Inflammatory markers measured in serum samples. Statistical significance is indicated as follows: * compared to the gel (vehicle) control group (p < 0.05); ^a^ compared to the negative control group (p < 0.05); ^b^ compared to the positive control group (p < 0.05); ^c^ compared to the CM-A30 group (p < 0.05); ^d^ compared to the CM-A60 group (p < 0.05); ^e^ compared to the CM-A90 group (p < 0.05); ^f^ compared to the CM-R5 group (p < 0.05); ^g^ compared to the CM-R10 group (p < 0.05). Measured parameters: a) IL-1β, b) IL-13, and c) TNF-α levels.

In the CM-containing gel groups (Group IV–IX), IL-1β levels were significantly decreased compared to the negative control group (p < 0.05) (Figure 4a). Among these, the CM-A30 and CM-A60 groups exhibited the most significant decreases in IL-1β levels and also showed a significant decrease compared to the positive control group (Figure 4a). IL-13 levels were markedly reduced by the CM-A30 and CM-A60 gel applications in comparison to the negative and positive control groups (p < 0.05) (Figure 4b). CM-A60 and CM-R15 gel applications significantly decreased TNF-α levels compared to both the negative and positive control groups (p < 0.05) (Figure 4c). Additionally, the CM-A30 group also showed a marked decrease in TNF-α levels compared to the positive control group (p < 0.05).

## 4. Discussion

New treatments continue to be investigated to improve delayed wound healing, which is one of the complications observed in diabetic patients. In this context, the dose-dependent healing effect of *Chelidonium majus* L. (CM), which has been reported to have wound healing potential in ethnobotanical studies, was investigated in vivo for the first time in diabetic wounds. In the study, gels containing different doses of aerial or root parts of the plant were applied to the diabetic wound model created in rats and their effects were determined.

One of the important physical indicators that a wound has started to heal is its closure as a result of reepithelialization. According to the WCR results obtained in our study, CM-containing gels showed the best effect at doses of 60 mg/kg (aerial parts) and 15 mg/kg (root). In their ethnobotanical study conducted in 2012, Kızılarslan and Özhatay (2012) reported that CM was used as a wound healing agent. Similarly, Kültür (2007) reported that CM was used folklorically for medicinal purposes in wounds and inflamed wounds. In another study, Mouro et al. (2021) showed that CM-containing nanofibrous meshes had potential as effective wound dressings by inhibiting bacterial infections in the healing process. Moreover, quercetin, one of the major phenolic compounds of the root part of CM, accelerates diabetic wound healing (Fu et al., 2020). Gallic acid, one of the major phenolic compounds of the aerial part of CM, improves wound healing by accelerating cell migration of fibroblasts and keratinocytes under both normal and diabetic circumstances (Yang et al., 2016). Similarly, berberine, one of the major alkaloids found in both the root and aerial parts of CM, increases wound closure and improves wound healing in diabetic rats (Zhou et al., 2021; Zhang et al., 2022). Considering these studies supporting our results, it can be stated for the first time that CM accelerates the healing process in an in vivo diabetic wound healing model. This effect varies according to the dose and the part of the plant used. These macroscopic observations were corroborated by the histopathological findings. Overall, the CM-containing gels and positive gel groups demonstrated superior histopathological results than the negative control group. These groups showed moderate reepithelialization, stronger granulation tissue, and enhanced angiogenesis and collagen organization. Therefore, CM-containing gels show significant potential for enhancing the healing process in diabetic patients.

Changes in oxidative events, disruption of oxidative balance in favor of oxidants, and protein, lipid, and DNA damage resulting from this disruption are important reasons for delayed wound healing in diabetics. Previous research has found that malondialdehyde (MDA) and PC levels are higher in diabetic or chronic wounds in contrast to normal wounds (Rattan and Nayak, 2008; Kaltalioglu et al., 2020). In our study, CM-containing gels significantly decreased MDA levels, a significant indicator of lipid peroxidation, compared to the negative control group. When PC levels indicating protein damage were examined, it was observed that only CM-containing gels at doses of 30 mg/kg (aerial parts) and 15 mg/kg (root) significantly reduced PC levels compared to the negative control group. While CM-containing gel application provided protection against lipid damage in all groups, its effect against protein damage was limited. This may have occurred due to the dose and duration of application. It has been reported that both aerial and root parts of CM have antioxidant activity due to the secondary metabolites they contain (aerial parts were found to be more effective than root) (Nile et al., 2021). In their study, Paul et al. (2013) reported that administration of chelidonine, a bioingredient of CM, significantly reduced lipid peroxidation activity (via MDA levels) and increased catalase, glutathione reductase, superoxide dismutase, and GSH levels in the liver tissue of cadmium-intoxicated mice. Similarly, CM extract restores the decreased GSH level and reduces increased NO in aflatoxin B1-induced hepatotoxic rats (Gomaa et al., 2022). Sinan et al. (2024) found that chelidonic acid, an organic acid component of CM, enhances GSH levels in both serum and hippocampus. GSH, a powerful antioxidant, plays an important role in defense against oxidative stress by reducing reactive oxygen species (ROS) with its antioxidant properties (Dindar et al., 2017). In our study, CM-containing gels at doses of 30 mg/kg and 60 mg/kg (aerial parts), and 10 mg/kg (root) significantly increased GSH levels compared to the negative control group. Nitric oxide, which is synthesized primarily by macrophages, endothelial cells, and fibroblasts in the wound area through the nitric oxide synthases (NOSs), is a highly reactive molecule and can cause nitrosative stress when found in high levels (Frank et al., 2002; Schwentker et al., 2002). In our study, CM-containing gels significantly reduced NOx levels compared to the negative control. Stylopine, a major compound of CM, reduces the production of NO in macrophages by suppressing the expression of inducible nitric oxide synthase (iNOS) in a concentration-dependent manner (Jang et al., 2004). Collagen and AA are essential components of diabetic wound healing. Collagen provides structural support for dermal wound healing. AA is required for collagen synthesis and is involved in antioxidant defense (Pullar et al., 2017; Maeso et al., 2024). In this study, CM-containing gels at doses of 30 mg/kg and 60 mg/kg (aerial parts) significantly elevated collagen and AA levels in parallel compared to the negative control group. In support of our findings, it has been reported that CM extract improves collagen synthesis by inhibiting elastase activity (Lee et al., 2006; Arora and Sharma, 2013). CM may have exhibited this effect due to its rutin (Arora and Sharma, 2013) content. Chen et al. (2020) reported that rutin improved collagen fiber proliferation during the wound healing process in diabetic rats. Our results indicate that CM application improves diabetic wound healing by contributing to the establishment of oxidative balance and collagen production due to the mentioned properties.

The proinflammatory cytokines TNF-α and IL-1β are primarily involved in the inflammatory process during wound healing. Diabetic wounds produce three times more TNF-α than normal wounds (Siqueira et al., 2010). It is also known that diabetes induces IL-1β expression in many different cell types, especially macrophages (Koenen et al., 2011). Considerable evidence suggests that increased inflammation due to overproduction of these cytokines may delay wound healing (Xu et al., 2013). In our study, all CM treatments decreased IL-1β levels, while TNF-α levels decreased depending on the dose and the part of the plant. These decreases may be due to the alkaloids present in CM. Zielińska et al. (2019; 2020) reported that the alkaloid components chelidonine, chelerythrine, sanguinarine, and berberine in both the root and aerial parts of CM reduced TNF-α and IL-1β secretion in human neutrophils. These results provide further support for the hypothesis that CM decreases proinflammatory cytokine secretion in a dose-dependent manner. Additionally, IL-13 is an antiinflammatory cytokine secreted by M2 macrophages, and its level is reduced in diabetic wounds. This is also thought to contribute to impaired wound healing (Aitcheson et al., 2021; Sheng et al., 2023). In our results, CM-containing gel applications had no effect on IL-13 levels in general, but only gels containing low doses of aerial parts showed an effect. This may be due to the chelidonine present in CM (Kim et al., 2015).

MMPs are found in both acute and chronic wounds. Together with their inhibitors, they serve a critical function in controlling extracellular matrix (ECM) breakdown and deposition, which is required for wound reepithelialization. MMP-2 and MMP-9 (gelatinases) play a vital role in keratinocyte migration and angiogenesis regulation in the healing process (Caley et al., 2015). However, chronic wounds are characterized by high MMP-2 and MMP-9 levels (Kandhwal et al., 2022). Previously, it was shown that MMP-2 and MMP-9 protein expression levels were significantly higher in diabetic wounds compared to nondiabetic wounds (Dai et al., 2021). In our study, CM-containing gels decreased MMP-2 and MMP-9 levels depending on the dose and part of the plant. Hong et al. (2020) found that CM extract reduces MMP-2 and MMP-9 production and activity in a dose-dependent manner. Considering the results, CM application may have contributed to wound healing by suppressing MMP-2 and MMP-9 production.

## Conclusion

This study demonstrates that different doses and parts of CM may improve diabetic wound healing by modulating key biochemical and histopathological markers. In particular, CM gels enhanced collagen deposition, which is essential for tissue integrity, and decreased levels of MMP-2 and MMP-9, which are enzymes linked to extracellular matrix degradation. Moreover, CM gel application reduced inflammatory cytokines and oxidative stress. The dose-dependent effects observed between the aerial parts and root extracts highlight the therapeutic potential of CM in tailoring wound care strategies for diabetic patients. The results of the study have important implications for the treatment of diabetic wounds, as they offer strong evidence that CM is a natural, plant-based treatment. In future studies, the primary objectives should be to optimize dosing regimens, elucidate the molecular pathways underlying the observed effects, and evaluate the long-term safety and efficacy of CM-based formulations.

## Figures and Tables

**Table 1:** Experimental group descriptions.

Group numbers	Groups	Specifications
Group I	Gel (Vehicle) control	The vehicle (blank) gel was topically applied to each wound for 7 days
Group II	Negative control	No product was applied to the wounds in the untreated group
Group III	Positive control	Madecassol, the reference drug, was topically applied to each wound for 7 days
Group IV	CM-A30	A gel containing 3% *C. majus* aerial part extract (30 mg/kg bw) was topically applied to each wound for 7 days
Group V	CM-A60	A gel containing 6% *C. majus* aerial part extract (60 mg/kg bw) was topically applied to each wound for 7 days
Group VI	CM-A90	A gel containing 9% *C. majus* aerial part extract (90 mg/kg bw) was topically applied to each wound for 7 days
Group VII	CM-R5	A gel containing 0.5% *C. majus* root extract (5 mg/kg bw) was topically applied to each wound for 7 days
Group VIII	CM-R10	A gel containing 1% *C. majus* root extract (10 mg/kg bw) was topically applied to each wound for 7 days
Group IX	CM-R15	A gel containing 1.5% *C. majus* root extract (15 mg/kg bw) was topically applied to each wound for 7 days

**Table 2:** Effects of CM-containing gel applications on wound closure rate in diabetic rats.

Groups	WCRs (%)
Gel (Vehicle) control	35.71 ± 3.65
Negative control	33.05 ± 3.38
Positive control	65.87 ± 3.87*,^a^
CM-A30	65.24 ± 3.06*,^a^
CM-A60	79.31 ± 1.76*,^a^,^b^,^c^
CM-A90	67.86 ± 2.00*,^a^,^d^
CM-R5	66.97 ± 2.59*,^a^,^d^
CM-R10	66.03 ± 2.54*,^a^,^d^
CM-R15	78.82 ± 1.67*,^a^,^b^,^c^,^e^,^f^,^g^

Statistical significance is indicated as follows:

* compared to the gel (vehicle) control group (p < 0.05);

a compared to the negative control group (p < 0.05);

b compared to the positive control group (p < 0.05);

c compared to the CM-A30 group (p < 0.05);

d compared to the CM-A60 group (p < 0.05);

e compared to the CM-A90 group (p < 0.05);

f compared to the CM-R5 group (p < 0.05);

g compared to the CM-R10 group (p < 0.05).
